# Assessing the effects of the nursing education reform on the educational environment in Tajikistan: a repeated cross-sectional analysis

**DOI:** 10.1186/s12912-020-0405-4

**Published:** 2020-02-11

**Authors:** Filippo Lechthaler, Michele Arigoni, Mohira Khamidova, Dilbar Davlyatova, Helen Prytherch, Kaspar Wyss

**Affiliations:** 1 Swiss Tropical and Public Health Institute, Socinstrasse 57, P.O. Box, 4002, Basel, Switzerland; 2 University of Basel, Basel, Switzerland; 3 School of Agricultural, Forest and Food Sciences, Bern University of Applied Sciences, Zollikofen, Switzerland; 4 Medical Education Reform Project, Dushanbe, Tajikistan; 5 Swiss Tropical and Public Health Institute in the Republic of Tajikistan, Shota Rustaveli 35, Dushanbe, Tajikistan

**Keywords:** Tajikistan, Health care reform, Medical education, Nursing students

## Abstract

**Background:**

A well-functioning education system for family nurses is a priority of the primary health care reform in Tajikistan. In 2015/2016, a baseline study was carried out to measure the educational environment at two nursing colleges, in Kulob and Dushanbe. Building on the study’s recommendations, the educational reform has addressed several key issues to improve the educational environment among nursing students with a focus on strengthening competency-based learning and clinical skills. A follow-up study was carried out in late 2018 to comparatively analyse progress in the educational environment against the baseline and assess potential impacts of tailored interventions.

**Method:**

A repeated cross-sectional survey involving 1239 students was applied to measure changes in the educational environment between 2015/2016 (baseline) and 2018 (endline) using the Dundee Ready Education Environment Measure (DREEM). We compared mean scores over time using Welch’s two sample t-test and the Wilcoxon-Mann-Whitney test. Single items have additionally been analysed using critical threshold (flags) for mean scores, and the percentage of answers falling above or below predefined values. A multivariate non-parametric regression was applied to control for confounding factors. Internal consistency was examined using Cronbach’s α.

**Results:**

Cronbach’s α for overall scores ranged between 0.87 and 0.89. Between 2015/2016 and 2018 the perceived educational environment improved with an increase of the mean total DREEM score from 131.8 to 146.9 in Dushanbe and from 134.9 to 151.2 in Kulob. Mean comparisons and multivariate regression revealed a significant increase of all sub-scores between 2015/2016 and 2018 with students’ social self-perception exhibiting the smallest progress. Despite the general improvements observed, analysis at the level of single items revealed persistent weaknesses including a lack of competency-based learning and stress.

**Conclusions:**

The education environment has improved in several important ways between 2015/2016 and 2018 which points to a likely positive contribution of the nursing education reform. This progress notwithstanding, there is still notable room for further improvement. Targeted efforts aimed at a better organization of practical trainings, improved didactical competences of teachers, and support structures for lonely and stressed students still seem to be lacking for the achievement of a good nursing education system in Tajikistan.

## Background

After its independence in 1991, Tajikistan decided to move from a highly specialized health system to one with family medicine as pillar of service delivery. The Ministry of Health and Social Protection of the Population (MoHSP) of Tajikistan has identified medical education reform of family doctors and family nurses as a key priority within the National Health Strategy 2010–2020 [1]. Family practitioners (nurses and doctors) are part of the primary healthcare system and are specialized in caring for the entire family regardless of age and gender. Various reform plans have already been implemented including the establishment of two chairs for family medicine at the Tajik State Medical University, and an updating of the nurse training curriculum . Nurse education is a pre-service training that takes place at several medical/nursing colleges throughout the country. All nurse students follow a common track for the first 3 years after which they can enrol in a fourth year to qualify as either a family nurse or a midwife [2]. Despite this political transition, progress in the medical education system was slow with the training of nurses not receiving sufficient attention [3, 4]. Indeed, an assessment of the educational environment at two nursing colleges in 2015/2016 revealed substantial problems, including insufficient clinical exposure during training, lack of nurse tutors as role models, and weak pedagogical competencies at the faculty level -with an overemphasis on factual knowledge [5].

It is generally acknowledged that the educational environment influences students’ satisfaction and learning behaviour [6–8]. Several authors have highlighted that students with a more favourable perception of the educational environment are more successful academically [7, 9–11]. Consequently, educational reforms that target the improvement of the educational environment have become common practice all over the world [12, 13]. Monitoring and evaluating change in the educational environment over the course of medical education reforms is key to identify areas in need of attention and assess progress over time. For this purpose, the Dundee Ready Educational Environment Measure (DREEM) – a validated inventory designed to measure the educational environment at medical schools, for graduates (interns and residents), as well as for nursing, dental and chiropractic students – has been used in various countries around the world [14, 15]. Despite its strengths and wide range of applications, different reviews also point to low validity of the DREEM scales highlighting the need for psychometric testing.

In Tajikistan, the nursing education reform between 2015 and 2018 was majorly directed by the findings of a baseline study conducted in 2015/2016 at two principal nursing colleges in Dushanbe and Kulob using the DREEM inventory [5]. By assessing the state of the educational environment, six main areas in need of action were identified, namely an over-emphasis on factual learning, a lack of teachers’ pedagogical skills, seemingly flawed examination practices, the absence of a support system for stressed students, tired stud ents, and a severe lack of basic infrastructure. The educationally reform in nursing education between 2015/2016 and 2018 was therefore designed to address the over-emphasis on factual learning and foster competency-based training. More specifically, practical Skills Labs were launched to practice proper procedures on manikins and dummies, as well as other training equipment. Furthermore, a tutorship program was started for 4th-year students for which skilled nurses were selected and trained as clinical tutors. 4th-year students are now better supervised and guided by these clinical tutors while working for 7 weeks in clinics and rural health centres. An exchange visit was organized for clinical tutors as well as for teachers at nursing colleges. Teachers’ didactical skills were further addressed in visits of international experts and through didactical trainings.

This study presents the results from a repeated cross-sectional survey using the DREEM inventory. After measuring the perceived educational environment in 2015/2016 (baseline) we conducted a second survey in 2018 (endline) using a new sample of students enrolled at the same nursing colleges, in Dushanbe and Kulob. Results served to monitor progress in the educational environment over time and to evaluate possible contributions of the tailored educational interventions implemented between 2015 and 2018. The suitability of the DREEM inventory for the evaluation of nursing education environment in a Tajik setting was assessed beforehand testing the internal consistency of the DREEM scores by applying Cronbach’s α [16, 17].

While the DREEM inventory has been widely used in cross-sectional settings for diagnostics purposes [14], different studies pointed to the benefit of using longitudinal designs to evaluate progresses in the educational environment over the course of specific reforms [18–20]. Whereas a few studies used the DREEM inventory to assess developments in the educational environment at medical universities over time, the present work presents a first attempt to conduct a corresponding study in a nursing context.

## Methods

The aim of this study was to measure and analyse changes in the perceived educational environment in two nursing colleges (Dushanbe and Kulob) between the years 2015/2016 and 2018 and to discuss possible contributions of the educational interventions to these changes.

### Survey design and sampling

We applied a repeated cross-sectional analysis using the Dundee Ready Education Environment Measure (DREEM) to quantitatively measure the educational environment for nursing students at the nursing colleges in Dushanbe and Kulob. The study tool used for the survey is described in the article presenting the results from the baseline study [5]. Apart from the standardized DREEM items, we used questions on participants’ age and sex, as well as an open question at the end on the perceived educational environment.

The study population are 2nd- and 4th-year nursing students at two nursing colleges (Dushanbe and Kulob). A total number of 1200 study participants (nursing students) was targeted, with 300 participants per nursing college and study year. Students were re-sampled in 2018 selecting different individuals than in 2015/2016. For both, the base- and the endline study, sampling took place in two steps: Firstly, an exhaustive list of all classes with the numbers of students per group was obtained from the two nursing colleges. Classes were then randomly sampled from the list. Secondly, paper-based questionnaires were distributed to all enrolled students in the selected classes. The data collection for the baseline study took place in December 2015 in Kulob and in February 2016 in Dushanbe and the endline study at both sites in November/December 2018. The names of the students were not collected, and each questionnaire was identified only by a unique identifier.

### The DREEM

The DREEM covers 50 statements (or items) which are answered by participants based on a five-point Likert-scales. These 50 items can be classed in five thematic areas (sub-scales) that describe the educational environment which include (i) students’ perception of learning, (ii) students’ perception of teachers, (iii) students’ academic self-perception, (iv) students’ perception of atmosphere, and (v) students’ social self-perception (see also [21]). The five-point Likert-scales capture students’ degree of agreement with each statement. Nine negative items must be scored inversely for analysis and interpretation. The translation of the English version of the DREEM into Tajik and Russian has been described by Schubiger et al. [5]: a combined technique was used employing three bilingual translators and two rounds of back translation into English for verification [20]. The resulting questionnaire was pre-tested before use.

### Data analysis

Data were analysed for two domains (Dushanbe and Kulob nursing colleges) with a focus on assessing the progress of the DREEM scores between 2015/2016 and 2018. Data analysis widely followed the guidelines by Swift et al. [21] for analysing and reporting of the DREEM. The calculated scores were assessed based on the aggregated DREEM measure (total score), the five sub-scales (sub-scores), and 50 individual questionnaire statements (scores for single items). Interpretation of the overall score as well as sub-scale scores was done according to McAleer and Roff [6] as shown in Table 8 in the Appendix.

To assess changes in the perceived educational environment we compared the means for total scores, sub-scores and individual items between 2015/2016 and 2018 using the independent samples T-test and the non-parametric Wilcoxon-Mann-Whitney test. *P*-values < 0.05 were considered statistically significant. As DREEM items typically have bimodal or skewed distributions [22], a central measure like the mean or the median will hide relevant information of a skewed or bimodal distribution such as a high proportion of negative and positive responses. Following Swift et al. [21] the 50 single items were therefore additionally evaluated using thresholds (or flags) which enable to find patterns that otherwise may fall through the cracks. The following thresholds were applied (after recoding negative questions): (i) The lower threshold for the mean score is 2, indicating areas that need particular attention. The higher threshold is 3, indicating particularly strong areas; (ii) Percentage of answers with “strongly agree/agree” is lower than 50%; (iii) Percentage of answers with “disagree/strongly disagree” is higher than 20%; and (iv) Percentage of answers “unsure” is higher than 30%.

Multivariate regression was applied to explore the association of multiple covariates with the total score and the five sub-scores. To evade distributional influences from the data, we applied a local linear non-parametric estimation of the regression function using the method of kernels [23]. Year of study (undergraduates from year 1 and 2, and 4th-year students), sex, location (Kulob and Dushanbe) and the year of the survey were included as explanatory variables.

The internal consistency of the DREEM scales was tested using Cronbach’s α. Data was entered into an Excel spreadsheet. 10% of the questionnaires were selected randomly and double-checked to ensure the quality of the data entry. As quality was found to be sufficient for this subset of data (<1% errors), the remaining questionnaires were not double checked. Data analysis was conducted using the statistical software *R*.

## Results

### Respondent profile

**Fig. 1:**
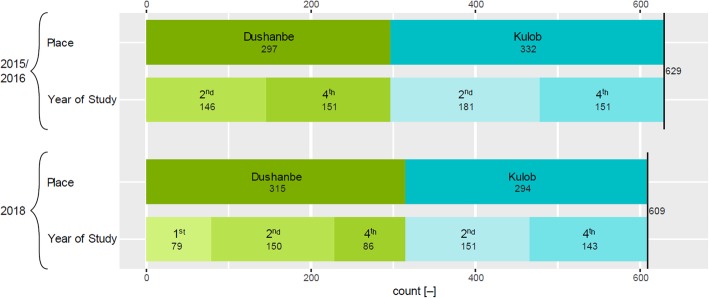
Breakdown of the study population for nursing college (place) and year of study. On the top the baseline study (2015/2016) and below the endline study (2018)

During the baseline study 629 questionnaires (72.8% female/27.2% male) and during the endline study 609 questionnaires (81.0% female/19.0 male) were filled out duly. Figure 1 shows the corresponding sample characteristics for the two studies, colleges and years of study. During the endline data collection many 4th-year male students were absent due to the endline data collection coinciding with the recruitment period for the Tajik army. In Dushanbe, a total of only 86 4th-year students could be questioned, and the questionnaire was therefore additionally distributed to 79 1st-year students to obtain a sufficient sample size and to improve representativity of the gender ratio. The students that participated in the endline survey are on average 1 year younger than the students that took part in the baseline study in both nursing colleges.

### Psychometric properties

The internal consistency has been assessed at the level of sub- and total scores using Cronbach’s α (Table 1). Except for the sub-score that captures students’ social self-perception, the corresponding values range between 0.607 and 0.749. Values for social self-perception was 0.302 in 2015/2016 and 0.041 in 2018. In general, all values for Cronbach’s α have slightly decreased between the baseline and the endline study. The values for the total scores was 0.891 in 2015/2016 and decreased to 0.873 in 2018.

**Table 1:** Cronbach’s α for the five sub- and the total score

	2015	2018
Students’ perception of learning	0.683	0.652
Students’ perception of teachers	0.607	0.658
Students’ academic self-perception	0.749	0.745
Students’ perception of atmosphere	0.715	0.664
Students’ social self-perception	0.302	0.041
Total Score	0.891	0.873

### Results at the level of sub- and total scores

All sub-scores and total scores for both colleges exhibited significantly higher mean scores in 2018 than in 2015/2016. Table 2 reports the corresponding estimates for both nursing colleges. In Dushanbe the mean total score has increased from 131.8 (65.9% of the maximum score) to 146.9 (73.4% of the maximum score) which represents a relative increase of 7.6% of the maximum score. The highest increase relative to the maximum score could be observed for students’ perception of teachers (10.3%) followed by students’ perception of atmosphere (7.7%), students’ academic self-perception (7.4%) and students’ perception of learning (7.2%). The sub-score for students’ social self-perception changed to a lesser extent with 3.9%. Changes in mean scores over time showed a similar pattern at the nursing college in Kulob. The mean total score has increased from 134.9 (67.5% of the maximum score) to 151.2 (75.6% of the maximum score) which represents a relative increase of 8.1% of the maximum score. The strongest increase was found for the sub-score describing students’ academic self-perception (9.8%) followed by students’ perception of teachers (9.4%), students’ perception of atmosphere (9.1%) and students’ perception of learning (6.7%). As for Dushanbe, students’ social self-perception (5.1%) developed to a lesser extent.

**Table 2:** Comparison of the total scores and sub-scores between 2015/2016 (baseline) and 2018 (endline) for the nursing colleges in Dushanbe and Kulob

		Dushanbe						Kulob					
		2015 (*n* = 297)		2018 (*n* = 315)		*p*-values		2016 (*n* = 332)		2018 (*n* = 294)		*p*-value	
Subscale	Max Score	Mean *(sd)*	% of max	*Mean (sd)*	% of max	t-test	WMW-test	Mean *(sd)*	% of max	*Mean (sd)*	% of max	t-test	WMW-test
Students’ perception of learning	48	31.2 *(6.0)*	65.0	34.7 *(5.3)*	72.2	<1e-04	<1e-04	32.1 *(5.5)*	67.0	35.4 *(4.5)*	73.7	<1e-04	<1e-04
Students’ perception of teachers	44	27.5 *(5.5)*	62.5	32.0 *(5.5)*	72.8	<1e-04	<1e-04	28.0 *(5.4)*	63.7	32.1 *(5.1)*	73.1	<1e-04	<1e-04
Students’ academic self-perception	32	22.6 *(4.7)*	70.6	25.0 *(4.4)*	78.0	<1e-04	<1e-04	23.7 *(4.8)*	74.0	26.8 *(3.5)*	83.7	<1e-04	<1e-04
Students’ perception of atmosphere	48	32.0 *(6.3)*	66.7	35.7 *(5.7)*	74.4	<1e-04	<1e-04	32.8 *(6.3)*	68.4	37.2 *(4.9)*	77.4	<1e-04	<1e-04
Students’ social self-perception	28	18.4 *(3.2)*	65.8	19.5 *(3.1)*	69.7	<1e-04	<1e-04	18.3 *(3.5)*	65.2	19.7 *(2.7)*	70.3	<1e-04	<1e-04
Total Score	200	131.8 *(21.3)*	65.9	146.9 *(19.1)*	73.4	<1e-04	<1e-04	134.9 *(20.6)*	67.5	151.2 *(16.3)*	75.6	<1e-04	<1e-04

### Results at the item level

In the following we describe changes in single items over time, focussing on reporting items that negatively stand out (mean values below 2 and percentage of answers with “disagree/strongly disagree” higher than 20%).

*Students’ perception of learning* (Table 3): In Dushanbe, for 9 out of 12 items the mean scores increased significantly between 2015 and 2018. Item 25 “The teaching over-emphasises factual learning” and item 48 “The teaching is too teacher-centred” did not increase significantly and additionally have mean values below the threshold of 2. In Kulob, 9 out of 12 items exhibited significantly higher mean scores in 2018. The mean scores for “The teaching over-emphasizes factual learning” (25) and “The teaching is too teacher-centred” (48) significantly decreased and were below the threshold of 2 in 2018.

**Table 3:**
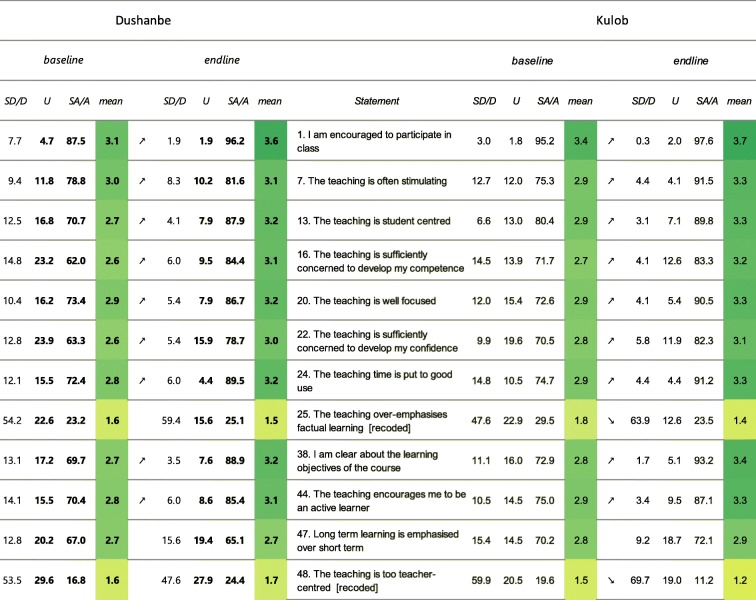
Results presented by “flagged items” for the sub-score “Students’ perception of learning”

Note: Flagged items are in grey and the number (s) causing the flagging are set in bold. The arrows show a significant in- or decrease. The colour is scaled to the mean score. Legend: SA/A: Strongly agree/Agree, U: Undecided, D/SD: Disagree/Strongly disagree, all in percentages

*Students’ perception of teachers* (Table 4): in Dushanbe, the items that constitute students’ perception of teachers showed a significant increase in mean scores in 10 out of 11 cases. The items 9, 32, 39 and 50 – i.e. “The teachers are authoritarian”, “The teachers provide constructive criticism here “, “The teachers get angry in class” and “The students irritate the teachers” – remained flagged with more than 20% of students providing low scores (0 or 1). In Kulob, the mean scores for 10 out of 11 items that constitute students’ perception of teachers have increased significantly between 2016 and 2018. The three items “The teachers provide constructive criticism here” (32), “The teachers get angry in class” (39) and “The students irritate the teachers” (50) remained flagged for a high percentage (> 20%) of students which provided low scores (0 or 1).

**Table 4:**
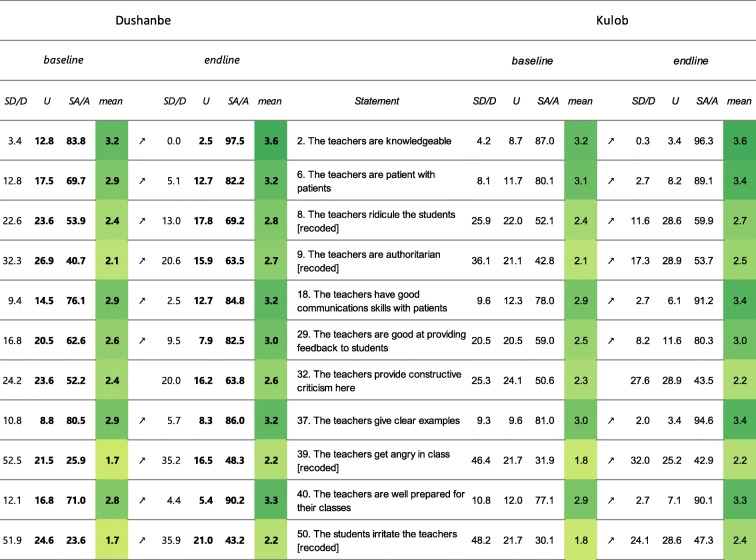
Results presented by “flagged items” for the sub-score “Students’ perception of teachers”

Note: Flagged items are in grey and the number (s) causing the flagging are set in bold. The arrows show a significant in- or decrease. The colour is scaled to the mean score. Legend: SA/A: Strongly agree/Agree, U: Undecided, D/SD: Disagree/Strongly disagree, all in percentages

*Students’ academic self-perception* (Table 5): in Dushanbe, 6 out of 8 items showed significantly higher mean scores in 2018 as compared to 2015. In Kulob, all mean scores of the 8 items have significantly increased between 2016 and 2018.

**Table 5:**
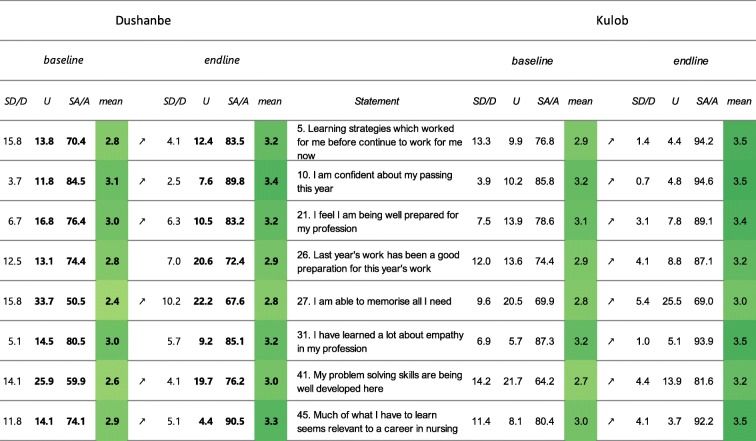
Results presented by “flagged items” for the sub-score “Students’ academic self-perception”

Note: Flagged items are in grey and the number (s) causing the flagging are set in bold. The arrows show a significant in- or decrease. The colour is scaled to the mean score. Legend: SA/A: Strongly agree/Agree, U: Undecided, D/SD: Disagree/Strongly disagree, all in percentages

*Students’ perception of atmosphere* (Table 6): in Dushanbe, the mean scores for 11 out of 12 items underwent a significant increase. Three items (“Cheating is a problem in this school” (17), “I find the experience disappointing” (35) and “The enjoyment outweighs the stress of studying nursing” (42)) remained flagged in 2018 for a high percentage of students that (strongly) disagreed (after recoding). In Kulob, 11 out of the 12 items that constitute the sub-score “Students’ perception of atmosphere” revealed significantly higher mean scores in 2018 as compared to 2016. Two items revealed deficiencies and remained flagged in 2018: item 35 “I find the experience disappointing” and item 42 “The enjoyment outweighs the stress of studying nursing” for a high number of scores 0 and 1.

**Table 6:**
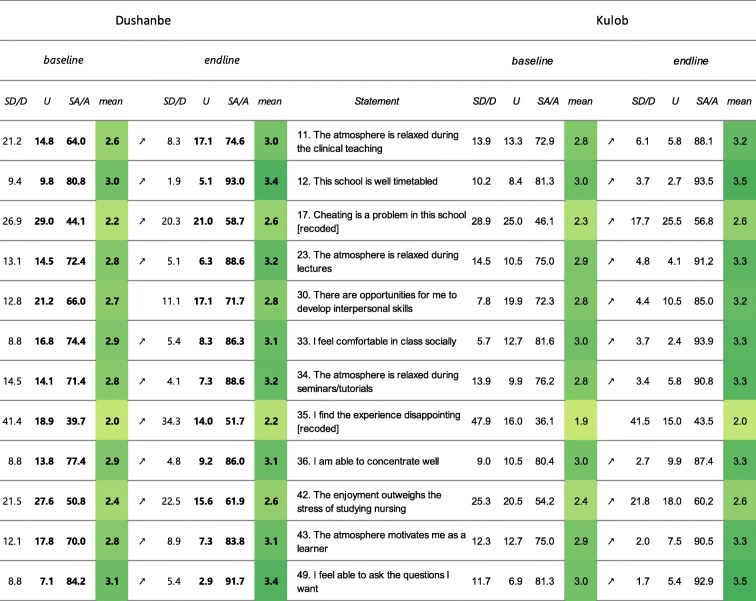
Results presented by “flagged items” for the sub-score “Students’ perception of atmosphere”

Note: Flagged items are in grey and the number (s) causing the flagging are set in bold. The arrows show a significant in- or decrease. The colour is scaled to the mean score. Legend: SA/A: Strongly agree/Agree, U: Undecided, D/SD: Disagree/Strongly disagree, all in percentages

*Students’ social self-perception* (Table 7): In Dushanbe, the scores of 5 out of 7 items significantly increased between 2015 and 2018. The items “There is a good support system for students who get stressed” (3), “I am too tired to enjoy this course” (4) and “I am rarely bored on this course” (14) remained flagged in 2018 with more than 20% of students providing low scores (between 0 or 1). The mean score for the item “I seldom feel lonely” (28) significantly decreased to a mean score below 2 in 2018. In Kulob, the mean score for 4 out of 7 items improved significantly between 2016 and 2018. The mean score of the item “I seldom feel lonely” (28) has significantly decreased between 2016 and 2018. Together with item (4) (“I am too tired to enjoy this course”), this mean score is below 2 in 2018. Item 14, “I am rarely bored on this course”, showed slightly lower values in 2018, although not significantly. The item remained flagged for a high percentage of students which (strongly) disagreed in 2018 (after recoding).

**Table 7:**
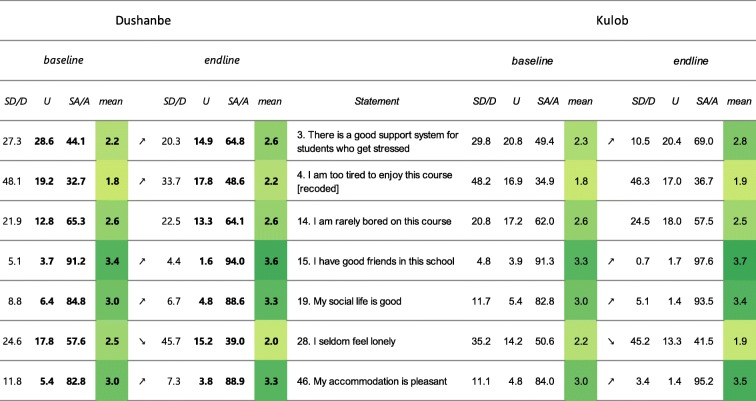
Results presented by “flagged items” for the sub-score “Students’ social self-perception”

Note: Flagged items are in grey and the number (s) causing the flagging are set in bold. The arrows show a significant in- or decrease. The colour is scaled to the mean score. Legend: SA/A: Strongly agree/Agree, U: Undecided, D/SD: Disagree/Strongly disagree, all in percentages

### Multivariate regression

**Fig. 2:**
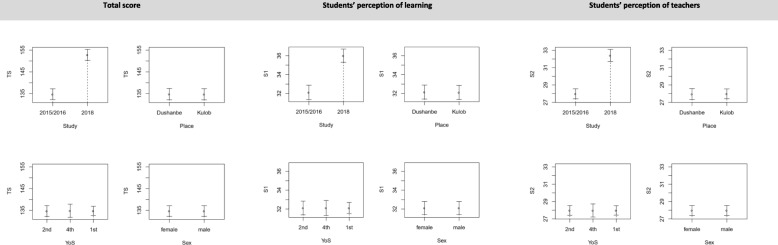
Partial local linear nonparametric regression plots Notes: TS: total score, S1-S2: sub-scores 1–2. Error bars are 95% bootstrapped confidence bands

**Fig. 3:**
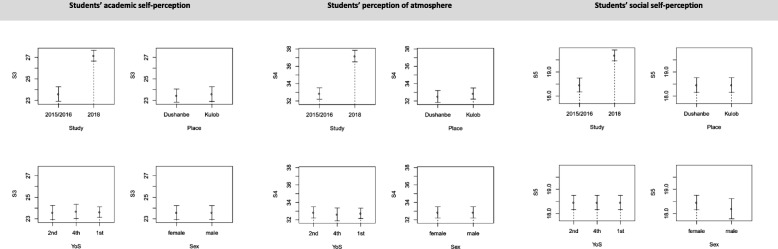
Partial local linear nonparametric regression plots Notes: S3-S5: sub-scores 3–5. Error bars are 95% bootstrapped confidence bands

Partial regression plots in Fig. 2 and Fig. 3 measured how strong the associations were between the DREEM scores and a single explanatory variable, controlling for other co-variates. Bootstrapped confidence bands (95%) are shown as error bars. Results revealed that, holding all other regressors constant at their mean, DREEM scores (total score and all 5 sub-score) were significantly higher in 2018 as compared to 2015/2016. Students’ social self-perception underwent the smallest increase (relative to the baseline) and students’ perception of teachers the highest, which mirrors the outcomes from the mean comparisons. All other covariates did not significantly explain any variation in the DREEM scores.

### Open question

In 2015/20156, most students’ comments (71.4%) were related to the lack of infrastructure. This issue appeared to be perceived less prominently by students in 2018 (9.4%). The second most important issue in 2015/2016 in relative frequencies were remarks about finance related issues constituting 27.2% of the comments. This fraction has as well de-creased to 5.9% in 2018.DISCUSSION.

For both colleges scores and sub-scores have increased between 2015/2016 and 2018. The differences are significant for all scores and sub-scores, and relative changes in mean scores are in the range of 3.9–10.2% of the maximum score. In Dushanbe the total score significantly increased from 131.8 to 146.9 which can be interpreted as a “more positive than negative” learning environment in 2018 according to the interpretation guideline presented in Appendix [6]. The score for the nursing college in Kulob showed the same tendency with a significant increase from 134.9 to 151.2. The education environment in Kulob can be interpreted as “excellent” in 2018. These results show that the educational environment for Tajik nursing students at two nursing colleges as measured by the DREEM inventory has reached above average levels in the international comparison. Indeed, with an overall mean of 146.9 in Dushanbe and 151.2 in Kulob, the mean score of this study was generally higher than other studies among nursing students in Chile (133.5) [24], China (132.5) [25], India (116.3) [26], Indonesia (132.0) [27], Malaysia (120.1) [28], Egypt (115.0) [29], and Iran (114.4) [30]. International comparison of DREEM scores, however, must be interpreted with care as the way participants’ respond to the questions is culturally sensitive [31] and scores do not only reflect differences in the actual educational environment.

Students’ perception of teachers, their academic self-perception, and their perception of the atmosphere underwent the largest changes between 2015/2016 and 2018, while students’ social self-perception changed the least. In 2015/2016, for both nursing colleges, the sub-score *students’ academic self-perception* achieved the highest relative score (in percent of the maximum), whereas *student’s perception of teachers* was the sub-score with the lowest value. In 2018, the sub-score *students’ academic self-perception* remained the highest relative score and *students’ social self-perception* instead of *students’ perception of teachers* achieved the lowest relative value for all subgroups. Indeed, the development of the faculties between 2015/2016 and 2018 was focused on fostering competency-based learning and more practical training through didactical trainings of teachers, the introduction of a Practical Skills Lab, and tutorship programs. The clear improvement in the perception of teachers, of atmosphere, of learning and on academic self-perception is likely to be associated with these increased and targeted efforts at the two nursing colleges. On the other hand, interventions were not focused at students’ social environment, which may mirror the smaller improvement in the social self-perception.

Analysis at the disaggregated item-level, however, allowed identifying specific issues within the sub-scores that have either stagnated or even decreased over time, constituting areas that need attention in the future development of the medical education system in Tajikistan. The survey in 2015/2016 revealed that students perceived the teaching as too heavily based on factual knowledge, which oriented the educational reform towards improving competency-based teaching and giving practical trainings more space in the curriculum. Surprisingly, the mean score of item 25 (“The teaching over-emphasizes factual learning”) further decreased between 2015/2016 and 2018. A possible reason for this result is that the 2018 survey took place before the clinical training of 4th-year students. Furthermore, students may have gotten aware of the benefits of competency-based learning so that they would like to see a further shift away from factual-learning approaches. Indeed, comments in the open question often mention the practical training positively but criticize that the actual exposure is insufficient – hence, there remains an urgent need to review current practices of competency-based teaching assuring that practical training and access to training equipment is adequately organized and equally distributed among students. Item 48 “The teaching is too teacher-centred” followed a similar pattern as item 25: despite the didactical trainings launched after 2015, the mean score did not improve significantly over time. Thus, further didactical trainings remain a priority to improve the teaching skills at faculty level. Furthermore, a structured assessment system providing regular feedback from the students to teachers (involving the faculty) would favor a more student-centered education environment.

Item 42 (“The enjoyment outweighs the stress of studying nursing”) exhibited a critical value in 2015/2016 and did not improve considerably during the intervention period. While there are several potential causes, stress can affect memory, concentration, and motivation ultimately leading to decreased learning and academic performance. Hence, there seems to be a continued need to monitor and evaluate potential causes of stress, while assuring that affected individuals are adequately supported.

Results from the open questions point to improvements in the teaching equipment and infrastructure.

Controlling for possible confounding factors in a multivariate regression design, DREEM scores revealed significantly higher values for 2018 as compared to 2015/2016. Other explanatory variables were not associated with the DREEM scores. Differences between gender were not statistically significant which is indicative for a discrimination-free environment.

With values for Cronbach’s α ranging between 0.60 and 0.90, overall internal consistency of the Tajik version of the DREEM is satisfactory, while the fifth subscale on students’ social self-perception is low (0.04 and 0.3). The low internal consistency of this score could be caused by culture-specific variations in response format [31]. The Tajik students’ life strongly differs from an average university in high-income countries. Tajik students seem to keep stronger ties to their families and are in some cases already married and have children. Being away from their families can lead to a feeling of loneliness (see item 28) while still having many good friends at school (see item 15). This disparity may lead to a comparatively low α-value indicating that the combination of items intended to measure social self-perception may not accurately capture the actual social context of Tajik nursing students. In general, there is no consensus over the cut-off level of Cronbach’s α for satisfactory scale reliability [32]. It has often been argued that a level of 0.70 is acceptable as described in Nunnally [33]. Others report values higher than 0.50 as being sufficient [25]. Wang et al. [25], using data for Chinese nursing students, report α values of sub-scales ranging from 0.62 to 0.90, and overall α of 0.95. O’Brien et al. [34], applying psychometric testing to the Singaporean version of the DREEM, report values ranging from 0.65 to 0.84 for sub-scales.

This study had limitations. Firstly, comparing students’ answers between 2015/2016 and 2018 based on a repeated cross-sectional design provides only suggestive evidence on the effectiveness of the corresponding interventions at the two nursing colleges. As the study design was not based on a controlled experiment, we did not have a valid counterfactual for comparing the educational environment in the absence of the interventions which causes potential biases in our estimates. Furthermore, the repeated cross-sectional design did not involve the observation of individuals over time which prevented the application of a panel regression model to control for unobserved heterogeneity in the study population. While we were able to control for possible confounding factors through the multivariate regression, the presented associations between the survey year and the educational environment are derived based on plausibility considerations and were not directly inferred from the analyzed data. Secondly, the general sample characteristics differed in some respects between the base- and endline study. The realized sample in Dushanbe in 2018 was augmented by including 1st-year students as 4th-year classes had high absence rate due to the military recruitment period. This led to differences in gender ratios and age distributions between the survey periods. Furthermore, as the interventions between 2015/2016 and 2018 focused primarily on the 4th year for family nursing and to a lesser degree on the earlier study years which may led to an overestimation of the potential effects of the interventions. Multivariate regression showed, however, that students’ year of study did not seem to significantly explain any variation in the DREEM scores. Thirdly, mean scores and sub-scores are slightly higher in Kulob as compared to Dushanbe during the base- and endline survey. A possible explanation is that students in Kulob were less critical and tended to respond more positively to the DREEM items. The investigation of possible reasons behind geographic disparities was beyond the objectives of the current study calling for more attention in future assessments. Fourthly, existing research shows that validity of the DREEM is not well supported [16, 17]. To control this risk, suitability of the DREEM inventory was statistically validated by testing for internal consistency. Lastly, participants’ responses to the items may be culturally sensitive [31]. Apart from translation, no cultural adaptation of the DREEM tool has been applied for the present study. This may represent are risk of bias and measured scores must therefore be interpreted with caution.

## Conclusions

The perceived nursing educational environment has improved between 2015/2016 and 2018 in Dushanbe and Kulob nursing colleges, suggesting that targeted interventions have positively contributed to a better learning environment. The relatively strong improvement in the perception of teachers, atmosphere, self-perception and learning are likely to be at least partly linked to specific tailored measures that aimed at fostering competency-based learning and practical training, such as didactical training of teachers, the strengthening of Practical Skills Labs, and the tutorship programs. On the other hand, there were only small improvements in the perceived social environment, which reflects the challenge to improve the social context through conventional educational reform programs. This study did not reveal any notable differences in the perceived educational environment between female and male students, which is indicative for a discrimination-free educational system. This progress notwithstanding, there is still notable room for further improvement. Targeted efforts aimed at a better organization of practical trainings, improved didactical competencies of teachers, regular assessments of teachers by students, and support structures for lonely and stressed students are needed to further improve the nursing education system in Tajikistan.

## Data Availability

The datasets used and analysed during the current study are available from the corresponding authors on reasonable request.

## Appendix

**Table 8:** Interpretation of the overall and sub-scale scores according to McAleer & Roff [6]

Total Score		Perception of learning	
0–50	Very poor	0–12	Very poor
51–100	Plenty of problems	13–24	Teaching is viewed negatively
101–150	More positive than negative	25–36	A more positive perception
151–200	Excellent	37–48	Teaching highly thought of
Perception of teachers		Academic self-perception	
0–11	Abysmal	0–8	Feelings of total failure
12–.22	In need of some retraining	9–16	Many negative aspects
23–33	Moving in the right direction	17–24	Feeling more on the positive side
34–44	Model course organisers	25–32	Confident
Perception of atmosphere		Social self – perception	
0–12	A terrible environment	0–7	Miserable
13–24	There are many issues which need changing	8–14	Not a nice place
25–36	A more positive attitude	15–21	Not too bad
37–48	A good feeling overall	22–28	Very good socially
